# Lived experiences of female second-cycle students with abnormal menstrual cycle in a selected municipality of Ghana: A phenomenological qualitative study

**DOI:** 10.1371/journal.pone.0345419

**Published:** 2026-03-25

**Authors:** Cate Degbor, Emmanuel Manu

**Affiliations:** Department of Population and Behavioural Sciences, Fred N.Binka School of Public Health, University of Health and Allied Sciences, Ho, Ghana; Cranfield University, UNITED KINGDOM OF GREAT BRITAIN AND NORTHERN IRELAND

## Abstract

**Background:**

Menstruation can be accompanied by stressful experiences and one such experience is the abnormal menstrual cycle, which could include menstrual irregularity and painful menstruation. Menstrual irregularity is defined as deviations in cycle length, frequency, or flow and can occur at any age, but it is most common among young women, often school-going children. Lack of understanding of abnormal menstruation often exposes young girls to adverse health outcomes. There is limited qualitative evidence on the lived experiences, beliefs, and coping strategies of secondary-school girls experiencing irregular menstrual cycles, particularly in rural settings.

**Objective:**

We explored the lived experiences of female second-cycle students with irregular menstrual cycles in a selected district in Ghana to inform policy on menstrual health education.

**Methods:**

We employed a phenomenological qualitative research approach to purposely interview 28 students using an in-depth interview guide. A thematic approach was used to analyze the data using ATLAS.ti v 7.5.

**Result:**

For lived experiences, patterns of menstrual flow characterized by inconsistent menstrual blood flow were a major reason they considered their menstrual cycle irregular. The challenges faced were physical and emotional. The students have personal beliefs that an irregular menstrual cycle is a sickness and a sign of infertility, which is caused by food and diet (unhealthy diet and sugary foods). The main coping mechanisms adopted were resting, hot water therapy, and medication and support from friends and family.

**Conclusion:**

The study provides an in-depth understanding of irregular menstrual cycles and their significant impact on the well-being of secondary school girls.

## Introduction

Menstruation, a monthly shedding of the uterine lining in reproductive-aged females [1,2] is a natural biological function experienced by over 1.8 billion girls and women globally [3]. Due to its nature, many women, especially adolescents, encounter challenges in managing this process. According to the American College of Obstetrics and Gynecologists (2021), 14 to 25% of women experience irregular cycles [4]. Irregular menstrual cycles are defined as menstrual patterns that deviate from the expected 21–35-day cycle, including frequent menstruation, prolonged or missed cycles, and unusually heavy or scanty bleeding [4]. Among adolescents, such irregularities may occur due to hormonal immaturity but can also signal underlying nutritional, psychosocial, or health-related challenges. These abnormalities often manifest as excessively heavy or light flow, cycle duration outside the 21 to 35 day range, or prolonged absence of menstruation [5]. Such irregularities are most prevalent among women under 23 years of age [6]. Most adolescents experience primary dysmenorrhea, which occurs without any underlying disease. However, some suffer from secondary dysmenorrhea, often caused by pelvic conditions like endometriosis, where the uterine lining grows outside the uterus [7]. About 500,000 adolescent girls worldwide feel frustrated during their periods due to complications from abnormal menstruation [8].

In Ghana, menstrual disorders including dysmenorrhea, menorrhagia, and premenstrual syndrome, are common among adolescent school girls and are associated with absenteeism, poor academic performance, and emotional distress [9]. Beyond academic implications, irregular menstrual cycles can affect the psychosocial well-being of young women and contribute to long-term health risks such as infertility and chronic conditions [7].

Menstruation is not only a biological event but also a social phenomenon which is often surrounded by stigma, cultural taboos, and misinformation [10]. In traditional African societies, including Ghana, menstruation is often viewed as unclean and is rarely discussed, limiting girls’ access to accurate information [11,12]. Education on both regular and irregular menstrual cycles equipped adolescent girls to recognize what constitutes a normal pattern and what may signal an underlying problem. This awareness empowers them to seek timely medical attention and appropriate support, thereby preventing complications such as anemia, reproductive tract infections, or infertility that may arise from untreated menstrual disorders [13,14]. Moreover, menstrual education promotes positive attitudes and coping strategies, reducing the stigma and misconceptions that often surround menstruation in many cultural contexts [11]. Girls who understand the biological, emotional, and social dimensions of menstruation are more likely to adopt healthy practices, manage pain effectively, and maintain good hygiene during their periods [11,15].

**Fig 1:**
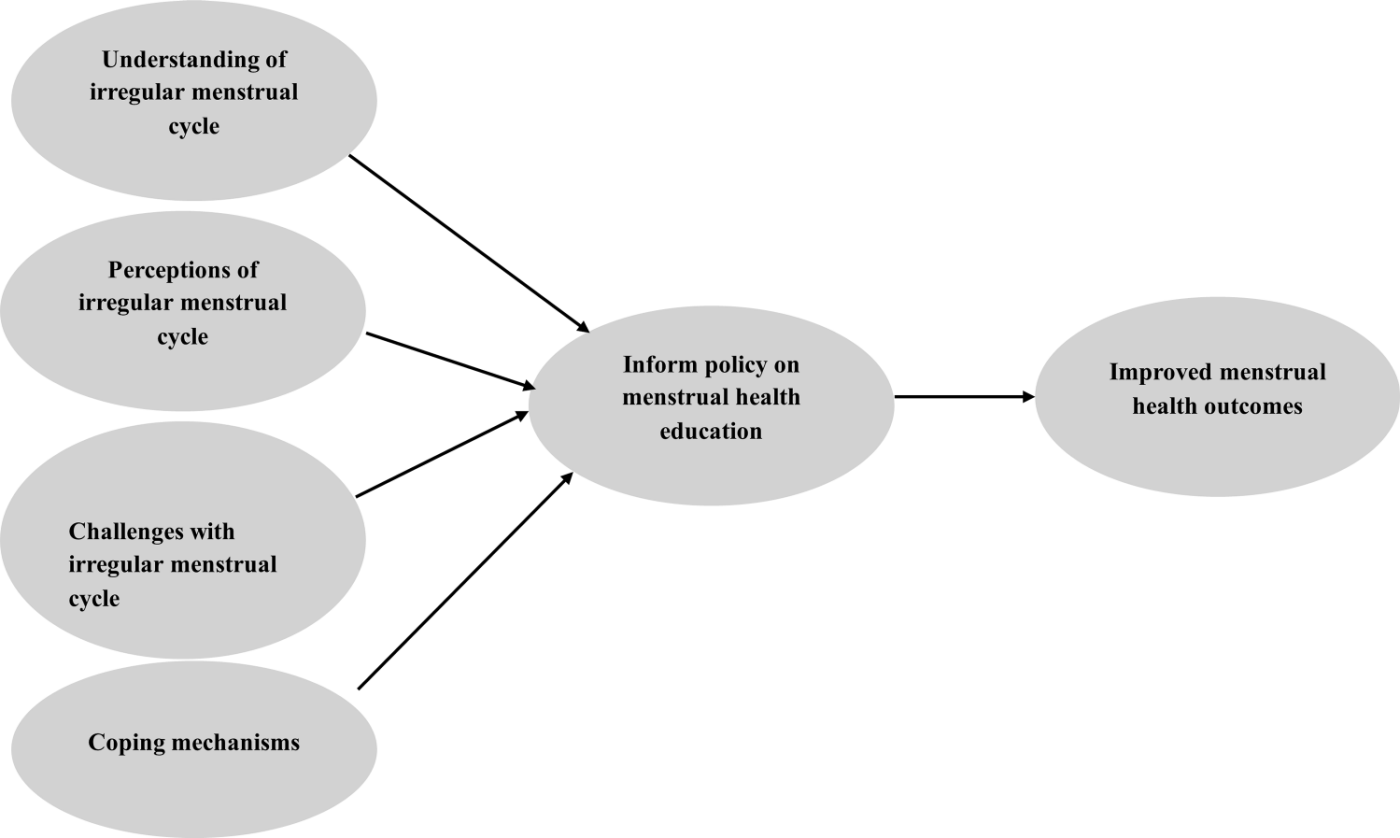
Conceptual framework.

Despite increased attention to menstrual health in low- and middle-income countries (LMICs), there remains a dearth of literature focusing specifically on the lived experiences of second-cycle female students in rural Ghana. Most existing studies focus on menstrual hygiene practices or access to sanitary products but fail to capture the nuanced emotional, cultural, and social dimensions of menstrual irregularities [16]. Furthermore, misconceptions such as equating early menstruation with loss of virginity or considering irregular menstruation a curse or sign of infertility still prevail in communities [17,18]. These beliefs can lead to shame, social exclusion, and reluctance to seek help, exacerbating the emotional trauma of irregular menstruation. Therefore, our study aimed to explore the lived experiences, beliefs, and coping mechanisms of female second-cycle students with abnormal menstrual cycles in a selected municipality in Ghana, per the tenets of the framework in Fig 1.

## Materials and methods

### Study setting

Our study was conducted in two selected Senior High Schools (SHS), also known as second-cycle schools, Bueman SHS and Okadjakrom SHS, in the Jasikan Municipality, located in the Oti Region of Ghana. The municipality has a population of approximately 59,695 [19]. It is predominantly rural and has three public SHS. The demographic landscape is characterized by a youthful population, with nearly 38% of residents under the age of 15, and a slight majority of females (50.8%). The Buem people are the primary ethnic group, and the economy is heavily dependent on agriculture, with key crops like cocoa and yams. Educationally, the municipality is served by a network of 85 kindergartens, 64 primary schools, 40 junior high schools, and 3 senior high schools, anchored by the Jasikan College of Education. Recent initiatives have focused on improving digital literacy by providing satellite internet to several schools. In terms of health, the Jasikan Municipal Hospital is the main referral facility, supported by 6 health centers and several smaller clinics. The study site was selected due to limited literature on menstrual health among adolescents in this locality.

### Study design

A descriptive phenomenological design was employed to explore the beliefs, experiences and coping mechanisms of SHS female second-cycle students in the Jasikan Municipality of Ghana, following the qualitative reporting guidelines of O’Brien at al. [20]. Descriptive phenomenology aims to understand the subjective lived experience of individuals as they occur naturally in real life settings [21]. This approach **was particularly suitable for the present study because it allowed the participants to share a narrative about their lived experiences on the subject.**

### Study population

The study was conducted among SHS female students with irregular menstrual cycles. The study included all female second-cycle students in two (2) selected SHS in the Jasikan Municipality who had either been medically diagnosed with an irregular menstrual cycle (e.g., abnormal frequency, duration, or flow) and students who self-reported any signs such as prolonged, irregular, painful, or heavy menstruation were included in the study. There were no age restrictions on participation. Students who met the inclusion criteria but felt uncomfortable being interviewed or were unwilling were excluded from the study.

### Researcher characteristics and reflexivity

The study was led by CD who was a Master of Public Health at the time of conducting the study. CD holds training in qualitative methods and has experience in menstrual and reproductive health research, and EM is a senior public health researcher with an extensive background in adolescent health. CD conducted the fieldwork and interviews, while EM provided oversight, guidance on data collection strategies, and support during analysis. CD is a young female public health researcher with experience in menstrual and reproductive health and advocacy perspectives. While this facilitated rapport and empathy with participants, it may have introduced assumptions that could influence data collection and interpretation. Additionally, her position as a researcher may have created a perceived power imbalance. EM’s extensive experience in adolescent health research could also have shaped the framing and interpretation of findings

Reflexivity was maintained at all stages of the study. The student researcher continually reflected on her role by **documenting** potential biases, recognizing how her background, personal experiences, and empathy toward participants could influence data collection and interpretation. Reflexive journaling was conducted after interviews to critically examine how CD’s positionality, expectations, and emotional responses may have shaped follow-up questions or preliminary interpretations. During analysis, coding decisions and theme development were discussed extensively between CD and EM to challenge assumptions and ensure that interpretations were grounded in participants’ accounts rather than prior beliefs or advocacy positions. The researchers did not know or were related to any of the study participants. To further eliminate interviewer biases, regular supervisory meetings between EM and CD were conducted to carefully review interview transcripts, coding, and emerging themes. These reflexive practices helped ensure that the findings accurately represented the participants’ lived experiences rather than the researchers’ preconceptions

### Sample size determination

Our study purposefully selected 28 female second cycle students with irregular menstrual cycle from two selected SHS (Bueman SHS and Okadjakrom SHS). Although data saturation [22] was reached at the 20^th^ participant, we continued interviewing up to 28 participants to ensure a broader representation across schools and to capture a wider range of experiences. Participants were recruited from 20/10/2024 to 03/12/2024 for data collection.

### Sampling procedure

At each school, all potential participants who met the inclusion criteria were purposefully selected. To identify eligible students, we organized health education sessions in each of the senior high schools to explain menstrual irregularities to the students, which include symptoms such as painful menstruation lasting less than three days, irregular flow, fatigue, and dizziness. The sessions were delivered by the research team and school health coordinators as general health education activities. Following the education sessions, students who self-identified or were referred by school health coordinators and teachers as having presented with such conditions were approached and those who met the inclusion criteria were selected.

### Data collection instruments and procedures

Data were collected through face-to-face in-depth interviews with an interview guide serving as an instrument. The interviews were conducted in either English or a Ghanaian language (Ewe), depending on the language preference of the students. The interviews were conducted within the school premises during school hours in a private and quiet space to ensure confidentiality. Each interview lasted for about 15 minutes which was appropriate considered the constraints of the school setting and the need to minimize disruption to instructional time The interview guide comprised 8–10 open-ended questions with probes, allowing participants to focus on the most salient aspects of their experiences within a time frame appropriate for a school setting. Both handwritten notes and audio recorders were used concurrently to document the interviews.

The interview guide was developed based on the study objectives, existing knowledge of menstrual health challenges, and discussions within the research team. The interview guide was structured into three sections. Section A captured participants’ socio-demographic characteristics (age, current form, ethnicity, religion and age of first menstruation), Section B focused on their understanding and beliefs about irregular menstrual cycles; and Section C explored the challenges faced, coping mechanisms used, and support systems available to them. Probes were used where necessary to gain a deeper understanding of participants’ experiences. To enhance the clarity and effectiveness of the data collection instrument, the interview guide was pre-tested among students in Hohoe Evangelical Presbyterian SHS who exhibited similar characteristics to the target population but were not part of the final study sample. The guide was reviewed by experts in qualitative research and adolescent health before pre-testing and refined based on feedback. Adjustments were also made based on the pre-test feedback to improve flow and comprehension. Interviews were conducted within the school environment at an isolated location which ensured privacy and comfort for the participants.

### Trustworthiness

To ensure trustworthiness of our study, we adhered to the Lincoln and Guba’s criteria [23]. To ensure credibility, rapport was established between the research team and school health coordinators prior to data collection. This helped create a safe and open environment where students could confidently share personal and sensitive experiences related to irregular menstruation. Additionally, experts in qualitative research were consulted to assist and review the instrument. Dependability was supported through the active involvement of trained qualitative researchers in both data collection and analysis. Peer debriefing and independent reviews of the transcripts and coding framework by qualitative research specialists helped to reduce researcher bias and ensure consistency in the interpretation of data. To ensure transferability, the study provides a rich description of the research setting, participant selection, data collection procedures, and analytical approach, allowing others to assess the relevance of the findings to similar contexts. Confirmability was ensured by engaging seven participants in the review of their individual transcripts and preliminary findings. This process validated the accuracy of the researchers’ interpretations and confirmed that the themes truly reflected the participants’ lived experiences.

CD led data collection, transcription, and preliminary coding. EM provided methodological oversight, independently reviewed transcripts and codes, and contributed to interpretation of findings. Peer debriefing involved experienced qualitative researchers external to the study team.

### Data analysis

Data collection and analysis were conducted simultaneously to allow for early identification of emerging themes. Each interview was transcribed verbatim and compiled into Microsoft Word files. Transcripts were then imported into ATLAS.ti version 7.5 for systematic coding. The data were analyzed using the descriptive phenomenological approach proposed by Colaizzi [24]. The analysis followed these seven steps: (1) Reading each transcript repeatedly to gain a holistic understanding of the data, (2) extracting significant statements directly related to the experience of irregular menstrual cycles, (3) formulating meanings from these statements while remaining faithful to the participants’ original words (4) grouping similar meanings into categories and identifying clusters of themes and overarching themes (5) developing a comprehensive description of the experiences shared by participants, (6) distilling the essential structure of the phenomenon under study, (7) conducting member checking by sharing transcripts and preliminary findings with seven participants for validation. Although themes were used to organize findings, they emerged inductively through phenomenological analysis aimed at capturing the essence of participants’ lived experiences.

To reduce bias and enhance the credibility of the findings, two members of the research team independently coded and analyzed the data. Regular team meetings were held to review emerging themes and resolve discrepancies. Where disagreements arose, a third independent was engaged solely to resolve coding disagreements. A theme was only accepted after consensus was reached. To ensure rigor, member checking was used not only for transcript verification but also for validating the researchers’ interpretation of participants’ experiences. Participants confirmed that the transcripts and identified themes accurately reflected what they shared during the interviews. Verbatim quotes from participants are included in the results section to further support the study’s interpretations.

### Ethical considerations

Ethical approval was obtained from the University of Health and Allied Sciences Research Ethics Committee (UHAS-REC A.8[43]23–24). Permission was also obtained from Jasikan District Education and the participating schools. Written and verbal informed consent was obtained from all participants, and child assent was obtained from students under 18 years with parental consent. Confidentiality was ensured through anonymization of data and secure storage of recordings. We ensured privacy by assigning codes to participants. The study was conducted in accordance with the ethical principles outlined in the Helsinki Declaration

## Results

A total of 28 participants were involved in the study. The majority, 25 (89.3%), were aged between 16 and 20 years. Most participants, 20 (71.4%), were in SHS 2. Over half, 16 (57.1%), were of Ewe ethnicity, and the vast majority, 26 (92.9%), identified as Christians. Most participants, 24 (85.7%), reported experiencing menarche between the ages of 10 and 15 (Table 1).

**Table 1:** Socio-demographic characteristics of the participants.

Variable	Frequency (n = 28)	Percent (%)
**Age**		
10 −15	3	10.7
16-20	25	89.3
**Current level**		
SHS 1	3	10.7
SHS 2	20	71.4
SHS 3	5	17.9
**Ethnicity**		
Ewe	16	57.1
Guan	6	21.4
Konkonma	4	14.3
Kotokoli	2	7.1
**Religion**		
Christianity	26	92.9
Muslim	2	7.1
**Age of Menarche**		
10-15	24	85.7
16-20	4	14.3

### Thematic findings

From the data we gathered, four (4) main themes emerged with respective sub-themes. These themes were understanding of irregular menstrual cycle (patterns of menstrual flow, signs and symptoms before menstruation), perception of irregular menstrual cycles (personal beliefs, perceived causes, societal norms), challenges faced by female students (physical challenges, social and emotional challenges, management barrier) and coping mechanism (stress management, support system, pain management). The themes and their corresponding sub-themes are presented in Table 2.

**Table 2:** Thematic findings from the participants.

Main theme	Sub theme	Sample quotes
Understanding of Irregular Menstrual Cycle	Patterns of Menstrual Flow	*Yeah, my menstruation experience was something like when I flow small then it will stop when I flow then it will stop” (ID 22, 16 years, SHS 2)*
	● Heavy flow	
	● Scanty flow	
	● Menstruating twice a month	
	● Inconsistent flow (heavy one day, light the next)	
	Signs and symptoms before menstruation	
	● Tenderness of breast	“*When I’m coming to have my menstruation, my abdomen starts spinning then, I feel tired. Every small thing I do, I feel tired, and I know that my menstruation is coming” (ID 34, 14 years, SHS1)*
	● Abdominal pains and menstrual cramps	
	● Loss of appetite	
	● Frequent urination ● General body weakness	
Perception of irregular Menstrual Cycles	Personal beliefs	*“I understand it’s like maybe I’m not fertile. I can’t reproduce, that’s how I understand it”* (ID 8, 17 years, SHS 2)
	● Menstrual irregularity is not a curse	
	● Sickness	
	● Infertility	
	Perceived causes	
	● Food and diet-related causes (sugary foods, unhealthy diet)	*“To my understanding, I think the way I used to eat sweets”* (ID 15, 19 years, SHS 2*).*
	Societal norms	
	● Menstruation starts at 18 for virgin	*“My mom told me that for her side, if you are a virgin, you will not menstruate until you are 18 years. At the age of 18, it will come by itself but if it comes before 18, it means you are no more a virgin* (ID 1, 17 years, SHS 2).
	● Don’t cook for men	
	● Don’t visit the river	
Challenges faced by female students	Physical challenges	
	● Excessive abdominal pains	*“At times when it comes like this week, I failed biology class. I will not be able to be permanent in class unless I stay outside or go to the sick bay to sleep”* (19years, SHS2)
	● Difficulty in learning	
	● Fatigue and Dizziness	
	● Absenteeism	
	● Difficulty in sleeping	
	Social and emotional challenges	
	● Annoyed by disturbance	*“And sometimes, you feel irritated. Like very much, when someone mentions your name, you will be pissed off. Kind of anger in you, trying to cope with the teacher, with your menses and things, it’s not easy”* (ID 2, 17 years, SHS 2)
	● Social withdrawal	
	● Emotional Stress (shyness, sadness, irritability)	
	Management barrier	
	● Lack of menstrual hygiene materials	*“Oh yes, I have some problems like that too. Like money, how to do and buy pads. Sometimes it’s something different”* (ID 25, 16 years, SHS 2)
	● Financial constraints	
Coping mechanism	Stress management	
	● Social avoidance	*“I don’t play. I don’t do any tedious work like that. I would just be sitting down quietly*” (ID 33, 17 years, SHS 2)
	● Resting	
	Support system	
	● Provision of sanitary pads and medicine by parent	*“In the school, sometimes my friends provide pads for me when I don’t have”* (ID 22, 16years, SHS 2)
	● Provision of sanitary pads by friends	
	● Assistance from friends in doing routine activity	
	Pain management	
	● Use of medication (painkillers)	*“In case the abdominal pain is severe, I normally take paracetamol to cool it down”* (ID 7, 16 years, SHS 2).
	● Hot water therapy for abdominal pain	
	● Social avoidance	

### Theme 1: Understanding of irregular menstrual cycle

From the perspective of SHS female students in the Jasikan district in the Oti region, an irregular menstrual cycle primarily refers to abnormalities or deviations from the expected pattern of menstruation. In the findings, two (2) sub-themes emerged: patterns of menstrual flow and signs and symptoms before menstruation.

#### Subtheme 1.1 Patterns of menstrual flow.

Regarding the patterns of menstrual flow, the students described it as a key indicator of abnormalities in their menstrual cycle. They explained that the irregularity is often characterized by differences in blood flow during their menstrual period. Specifically, they noted that the heavy flow characterizes irregularity, and mostly the inconsistent flow of the blood thus menstruating for two days, stopping on the third, and resuming later), or fluctuating patterns where the flow might be heavy one day, scanty the next, and normal thereafter. Some students had this to say. These are some quotes from the students

*“Yeah, my menstruation experience was something like when I flow small then it will stop, when I flow then it will stop*” (ID 22, 16 years, SHS 2).

*“For example, in a month, I can menstruate 2 times and sometimes too, I’ve been getting it the whole month”* (ID 4, 17 years, SHS 3).

#### Subtheme 1.2: Signs and symptoms.

Concerning signs and symptoms experienced before menstruation, the students reported a variety of physical and emotional changes that served as indicators of their upcoming menstrual cycle. Physically, they frequently mentioned breast tenderness, abdominal pain, and menstrual cramps as the most common signs. Other reported symptoms such as headaches, fatigue, and general body weakness, which often interfered with their ability to concentrate in class or participate in school activities.

For instance, they had this to say regarding the signs and symptoms.

*“When I’m coming to have my menstruation, my abdomen starts spinning then, I feel tired* (ID 9, 18 years, SHS 2)

*“I feel headache, very severe, my breast will be heavy and then my waist will be paining me as well as my abdomen”* (ID 5, 18 years, SHS2)

### Theme 2: Perception of Irregular Menstrual Cycles

We also identified the perception of female students regarding irregular menstruation, thus personal beliefs, perceived causes, and societal norms.

#### Subtheme 2.1 Personal beliefs.

About personal beliefs, some believe that abnormality was a sign of infertility, and most believe that it was sickness. However, most of them clearly distinguished menstrual irregularity from traditional or spiritual causes, emphasizing that it is not a curse or a result of wrongdoing..

*“I understand that it’s a sickness”* (ID 33, 17years, SHS 2)

*“I understand it’s like maybe I’m not fertile. I can’t reproduce, that’s how I understand it”* (ID 8, 17 years, SHS 2)

#### Subtheme 2.2 Perceived causes.

In relation to perceived causes, many students identified food and diet-related as the main causes of their irregularity, particularly the consumption of sugary foods, unhealthy diets, or a lack of balanced nutrition. They emphasized eating an unhealthy diet result in irregular menstruation. Here are some quotes from the students

*“To my understanding, I think the way I used to eat sweets”* (ID 15, 19 years, SHS 2*).*

*“I can’t really tell but what the doctor told me is I shouldn’t be keeping urine for a long time. I should decrease the level of sugary foods. I should eat more fruits and stuff”* (ID 13, 15 years, SHS1)

#### Subtheme 2.3: Societal norms.

Regarding irregular menstruation, the student reported some misconceptions such as menstruation starting at 18 years for virgins, they were not allowed to cook for men, and not visit the river. They explained that it has been explained to them that girls in their menstrual period are not to serve men, especially the elderly with food or assist them with anything.

*“My mom told me that for her side, if you are a virgin, you will not menstruate until you are 18 years. At the age of 18, it will come by itself but if it comes before 18, it means you are no longer a virgin* (ID 1, 17 years, SHS 2).

*“Yeah, the only taboo in my society is when you get your menses, example like my grandfather, you don’t fetch water for him to drink, you don’t cook for him, you don’t go close to his farm, and you separate your things from my grandmother, you don’t use her things to cook, else when you disobey his rules, the menstruation will not stop, until you tell him”* (ID 4, 17 years SHS 3).

### Theme 3: Challenges faced by female students

From the study, several challenges emerged which were classified under three sub-themes: physical challenges, emotional and social challenges, and management barriers.

#### Subtheme: 3.1 Physical challenges.

From the student abdominal pain emerged was one of the most severe challenges. They also reported felling fatigue, dizziness which eventually lead to absenteeism difficulty in learning and sleeping. Some students had this to say.

*“I face a lot of challenges when my menses come at midnight, I’m unable to sleep again because of the pain. Last two months when the pain came, I overdosed on drugs because the pain was unbearable and I fell sick for the whole week”* (ID 7, 16years, SHS 2).

*“At times when it comes like this week, I failed biology class. I will not be able to be permanent in class unless I stay outside or go to the sick bay to sleep”* (19years, SHS2).

#### Subtheme 3.2 Social and emotional challenges.

For social and emotional challenges, this subtheme captures the psychological and interpersonal difficulties students face during their menstrual cycle. They expressed feeling annoyed by disturbance, when dealing with physical discomfort like abdominal pain. Additionally, students experienced significant emotional stres**s**, such as feelings of shyness sadness and irritability. This mostly led to social withdrawal. The following quotes summarize their response.

*“I feel sad when I see my colleagues menstruating because I feel like maybe I can’t menstruate and some of them are laughing at me that maybe I can’t give birth and those things. I’m not fertile, that’s my mine is not coming”* (ID, 17 years, SHS2).

*“And sometimes, you feel irritated. Like very much, when someone mentions your name, you will be pissed off. Kind of anger in you, trying to cope with the teacher, with your menses and things, it’s not easy”* (ID 2, 17 years, SHS 2).

#### Subtheme 3.3: Management barriers.

With regards to the management barriers, the students reported lack of menstrual hygiene materials and financial constraints. The students noted financial challenges mostly prevented them from consistently obtaining sanitary pads.

*“Oh yes, I have some problems like that too. Like money, how to do and buy pads. Sometimes it’s something different”* (ID 25, 16 years, SHS 2).

*“When I am in pain like that, I do take a painkiller to reduce the pain for me. But when I am in school, because of financial problems I don’t get the money to buy it”* (ID 29, 18 years, SHS 2).

### Theme 4: Coping mechanisms

In our study, coping mechanisms were the ways student adopt to deal with challenging concerning irregular menstruation. Under this main theme, several coping mechanisms emerged which were grouped under three sub-themes.

#### Subtheme 4.1 Stress management.

With stress management, according to the students, they had different ways of coping with the stress associated with menstrual cramps due to their irregular menstruation. They mentioned the method of social avoidance where they isolate themselves from their friends. They also reported resting or sleeping to cope with the stress due to the physical pains. The following quotes summarizes their response;

*“I don’t play. I don’t do any tedious work like that. I would just be sitting down quietly”* (ID 33, 17 years, SHS 2)

*“Sometimes when it’s paining me, I just must take some rest then. It will be normal a bit”* (ID 34, 14years, SHS 1).

#### Subtheme 4.2 Support system.

The support system was the various forms of assistance students receive during menstruation. One common source of support was the provision of sanitary and medicine by parents. Similarly, the provision of sanitary pads by friends was frequently mentioned particularly in school where their friends support them with menstrual materials. They also emphasized assistance from friends in performing routine activities.

*“In the school, sometimes my friends provide pads for me when I don’t have”* (ID 22, 16years, SHS 2)

*“They’ll help me to bathe that’s all. My friends will send me to the bathhouse because walking becomes a problem for me. So, they fetch water for me, and I’ll bath, they’ll help me to walk on campus maybe when it’s prep time”* (ID 27, 18 years, SHS 2)

#### Subtheme 4.3: Pain management.

For pain management, the student reported using various strategies such as over-the-counter pain medication, particularly paracetamol, as well as non-pharmacological remedies like applying hot water to the abdomen. Some also practiced social avoidance to rest and manage discomfort in private. They noted that this method was easy and often seen as a natural and accessible way to ease cramps and promote relaxation. Some student had this to say;

*“In case the abdominal pain is severe, I normally take para to cool it down”* (ID 7, 16 years, SHS 2).

*“At home, I have been using hot water to warm my abdomen”* (ID 33, 17 years, SHS 2)

#### Essence of participants’ lived experiences.

Across all themes, the lived experience of irregular menstruation was characterised by uncertainty, distress, and disruption. Students described their menstrual cycles as unpredictable and frightening, often interpreting irregular bleeding as a sign of illness or future infertility. Physical pain, emotional instability, and academic interference were experienced simultaneously. In the absence of formal school-based health support, students relied heavily on peers, family members, and self-care strategies, reflecting both resilience and systemic gaps in adolescent menstrual healthcare.

## Discussion

This was qualitative study which explored the lived experiences, beliefs, and coping mechanisms of secondary school girls with irregular menstrual cycles. They were were predominantly aged 16–20 years and mostly in SHS 2, a stage often characterized by increasing academic pressure and psychosocial vulnerability. This developmental stage likely shaped how participants experienced menstrual irregularities, particularly the strong links between physical symptoms, emotional distress, and school functioning observed across theme**s**. Four (4) main themes emerged which were understanding of irregular menstrual cycle, challenges faced by female students, perception of irregular menstrual cycles, and coping mechanisms.

Our study found that the understanding of irregular menstrual cycles among secondary school girls is primarily shaped by the patterns of menstrual flow and the challenges they face. From our study, irregular menstrual patterns were often characterized by inconsistent flow, including heavy or scanty blood flow, which contributed significantly to the perception of abnormality. These findings align with existing definitions of irregular cycles, particularly oligomenorrhea, as described in the literature [25,26]. For instance, Riaz and Parekh (2023) define oligomenorrhea as an inconsistent menstrual blood flow, considered normal only in specific contexts such as the postpartum period or perimenopause. These conditions are often linked to underlying medical issues or poor dietary habits [27]. This implies that students experiencing irregular menstrual patterns may face complications such as infertility, endometrial hyperplasia, and a reduced quality of life due to the physical, emotional, and social burdens associated with irregular cycles [28]. These challenges may also hinder their academic performance and daily routines. These findings reflect that adolescent girls’ limited understanding of menstrual irregularities may delay appropriate health-seeking behaviour and increase their risk of long-term reproductive complications. Schools and health professionals therefore need to provide structured menstrual health education that clearly explains what constitutes normal and irregular menstrual cycles, as well as when to seek medical attention. Integrating menstrual health into school curricula and organizing periodic health talks by school nurses could improve awareness and early management of menstrual disorders.

We also identified physical, social emotional, and management barriers as the major challenges students faced. Physical challenges included severe abdominal pain, fatigue, and dizziness, which often resulted in difficulty concentrating and negatively impacted students’ academic performance due to absenteeism. These findings are consistent with studies [29,30], which reported that menstruation poses significant physical challenges for adolescent girls, interfering with their ability to attend school and fully participate in classroom activities. Additionally, heavy menstrual bleeding, often associated with fatigue due to blood loss, has been linked to a decrease in quality of life [31]. With management barriers, limited access to menstrual hygiene products and difficulty resting or coping during school hours were identified. This aligns with [32,33], which stated majority of schools are poorly prepared for menstrual hygiene lacking menstrual materials for emergencies and inadequate hygiene facilities. Dysmenorrhea, or menstrual pain, was identified as the most common menstrual challenge among adolescents [34], further highlighting the physical pain of menstruation. These findings reflect that menstrual health issues are not only medical concerns but also educational and psychosocial challenges that require a holistic response. Schools must therefore integrate menstrual health management into existing adolescent health programs to minimize absenteeism and academic disruption.

Our study also found social and emotional challenges, such as stress and social withdrawal. Emotional stress was characterized by feelings of shyness, sadness, and irritability, which often led to social withdrawal and reduced social engagement. These findings align with prior studies [35], where irritability and social isolation were frequently observed among adolescents. Similarly, [36,37] reported that students often experienced shame, guilt, fear, shyness, and sadness related to menstruation. These challenges may have significant implications for students’ mental health, potentially leading to psychological issues related to menstruation [38]. The implication of this finding is that menstrual health interventions should extend beyond physical management to include emotional and psychological support. Schools should provide safe, confidential spaces where girls can express their feelings and receive guidance from counselors or trained teachers.

Our study also revealed that students held diverse beliefs about menstrual irregularities, which were influenced by their personal beliefs, perceived causes, and societal norms. Regarding personal beliefs, many students perceived irregular menstruation as a sickness and an indicator of infertility. This belief aligns with findings by [39,40], which reported that irregular menstrual cycles are often associated with infertility issues. Several students expressed concerns about their ability to conceive in the future, attributing these fears to the irregularity of their cycles. However, most students rejected the idea that irregular menstruation is a curse, which reflect a shift from traditional stigmas and superstitions commonly associated with menstruation. These beliefs may attributed to lack of access to accurate menstrual health education, leading to misconceptions about the connection between menstrual irregularities and fertility [16,41]. The implications of these findings are significant, as these beliefs can contribute to emotional distress, reduced self-esteem, and heightened anxiety about future reproductive health.

Regarding perceived causes, students frequently stated food and diet as causes of menstrual irregularities, particularly the consumption of sugary foods and beverages. Our finding aligns with a study by [42–44], which found that junk food consumption negatively impacts menstrual cycles. Additionally, sweetened beverages have been identified as a risk factor for irregular menstruation. Armour et al. (2019) further highlighted the role of poor nutrition and lifestyle choices in contributing to menstrual problems and a decline in quality of life [45].

These perceptions may result from students’ intuitive understanding of the relationship between health and diet, and the widespread availability of sugary and processed foods, especially in school environments, could also play a role in shaping these beliefs [46]. The implications of these findings emphasize the importance of promoting healthy dietary habits among students. Schools and health professionals should implement educational programs that explain the relationship between lifestyle choices, nutrition, and menstrual health [27].

Our study found that students employed several coping mechanisms to manage the challenges associated with irregular menstrual cycles. We found strategies such as pain management, such as the use of medication (painkillers) and hot water therapy, and resting during episodes of pain, and seeking support from parents and friends. However, limited access to health facilities in schools affects effective management. The use of painkillers and hot water therapy is consistent with findings from studies [13,47], which reported that adolescents mostly rely on self-care methods to manage menstrual pain. Similarly, resting as a coping mechanism aligns with a study by Ní Chéileachair (2022), which found that students often rest in their beds to alleviate menstrual discomfort [48]. The reliance on social support is also supported by van Eijk et al. (2016), who highlighted the importance of peer and familial support in helping adolescents navigate the challenges of menstruation [49].

Self-care strategies and social support may result from limited access to healthcare services and menstrual products. While these coping mechanisms provide students with immediate relief, there are potential risks associated with frequent use of painkillers, such as dependency or side effects, if used improperly. These findings imply that while students demonstrate resilience in coping with the challenges of irregular menstrual cycles, the lack of adequate healthcare access and financial resources could lead to negative health outcomes. There is a need for comprehensive education on safe menstrual management practices, along with improved access to affordable healthcare and menstrual products.

### Future research directions

This study provides an in-depth understanding of how female second-cycle students experience and interpret irregular menstrual cycles within a rural Ghanaian context. However, further research is required to strengthen the evidence base for policy and programming. Future studies should adopt mixed-methods or longitudinal designs to examine how menstrual irregularities evolve over time and how early experiences influence long-term reproductive health outcomes, school retention, and psychosocial well-being. Quantitative studies involving larger and more diverse populations across multiple regions of Ghana would help determine the prevalence and correlates of abnormal menstrual cycles and allow for national-level policy planning.

### Limitations of the study

The study has several limitations. Participants were purposively selected from only two senior high schools in one municipality, which limits the generalization of the findings to other regions. The study also relied on self-reported experiences, which may have been influenced by recall bias or social desirability. However, the qualitative approach provided rich, in-depth insights into the lived experiences of students with irregular menstrual cycles.

A key limitation of this study is the relatively short duration of the interviews (approximately 15 minutes), which was necessary due to time constraints within the school setting. Although efforts were made to ask focused and probing questions, the limited time may have restricted the depth of exploration of participants’ experiences. Future studies could consider conducting longer interviews outside instructional hours or incorporating follow-up interviews to allow for more in-depth engagement and richer data generation.

## Conclusion

Our study provides an in-depth understanding of irregular menstrual cycles and their significant impact on the well-being of secondary school girls. There is a need for collaboration between Ghana Health Service and Ministry of Education to mitigate these challenges by improving access to healthcare services in schools, menstrual hygiene materials, and education on menstrual health. Strengthening coping strategies, such as enhancing social support systems and providing stress management resources, is essential to help students manage their cycles effectively.

## Supporting information

S1 File Data collection instrument. (DOCX)
