# Supplementary material for: Lived experiences of female second-cycle students with abnormal menstrual cycle in a selected municipality of Ghana: A phenomenological qualitative study
Source: PLoS One. 2026 Mar 25;21(3):e0345419. doi: 10.1371/journal.pone.0345419 (PMC13016279; doi:10.1371/journal.pone.0345419)
Supplement: S1 File — (DOCX) [file pone.0345419.s001.docx]

# **Data Collection Instrument [Interview Guide]**

SECTION A: SOCIO-DEMOGRAPHIC CHARACTERISTICS

How old are you?

Which form are you in?

What is your ethnicity?

What is your religion?

SESSION B: Female students’ understanding of irregular menstrual cycles.

1. Can you tell me about your first menstrual experience?

*Probe: What were your experiences like?*

1. How old were you when you had your first menstruation?
2. Was your first menstruation normal or you have experienced some experiences that made it different from normal?

*Probe: describe how the experience was (pain, irregularity, heavy flow, scanty flow)*

1. What are some of the signs and symptoms to experience that indicate to you that you are about to have your menstruation?
2. Are these symptoms persistent throughout your menstruation?
3. How do you understand the irregularity of your menstrual cycle? *Prob for: perceived cause, where they learn that understanding from*

SESSION C: The challenges, they face with the condition of abnormal menstrual cycles.

1. How do you manage your abnormal menstruation?
2. What are some of the challenges you face in the management of your abnormal menstrual cycle when in school and when in the house?

*Probe for: Final challenges, lack of social support, lack of a place to rest when in pain in school*

1. What are some of the menstrual hygiene practices you practice when menstruating?
2. What are some of the materials you use in managing yourself in a hygienic way?
3. How do you dispose of your used menstrual materials?
4. Why do you choose to dispose of the used material in that way?

SESSION D: Female students’ perception of abnormal menstrual cycles.

1. How do you perceive your abnormal menstrual cycles?
2. What do you think is responsible for your abnormal menstruation?
3. Do you believe your abnormal menstruation is a curse?

*Probe for the reasons for thinking so*

1. Do you believe your abnormal menstruation is caused by someone or God to punish you?
2. *Probe for the reasons for thinking or believing so*
3. Have you had any education on your abnormal menstruation?

*Probe for the type of education received*

1. What are the sources of your education?
2. Between home and school, where do you prefer to have your menses?
3. Why that place of preference?
4. What do you think can help improve your menstrual hygiene practices both in the house and in school?

**SESSION E: Female students coping mechanisms towards their conditions.**

1. How do you cope with stressful experiences that are associated with your menses?

*Probe for: Avoidance coping, religious coping, physical exercise, resting etc.*

1. Where do you learn those coping mechanisms from?
2. Have those coping strategies you mentioned effective for you in dealing with stressful experiences?
3. What support do you get during your menstrual cycle?

Probe: when in school [Emotional support, material support etc]

1. Who provides the type of support you mentioned?
2. Are you satisfied with the kind of support provided to you?
3. What do you suggest, can be done better to support adolescent girls in your school?
4. What support do you get during your menstrual cycle?

Probe: when in the Home or the community [Emotional support, material support

etc]

1. Who provides the type of support you mentioned when home?
2. Are you satisfied with the kind of support provided to you by your relatives and the community?
3. What do you suggest that can be done better to support adolescent girls with abnormal menstruation in schools and at home?
4. Are there any socio-cultural beliefs (taboo) regarding menstruation in your community?

Is there anything you would like to discuss with me about your menstruation and your experiences?
